# Supplementary material for: The Development of a Gleason Score-Related Gene Signature for Predicting the Prognosis of Prostate Cancer
Source: J Clin Med. 2022 Dec 1;11(23):7164. doi: 10.3390/jcm11237164 (PMC9737657; doi:10.3390/jcm11237164)
Supplement: Supplementary file 1 [file jcm-11-07164-s001.zip › jcm-2033759-supplementary.pdf]

Journal of
Clinical Medicine

MDPI

Supplementary Materials

The Development of a Gleason Score-Related Gene Signature for Predicting the Prognosis of Prostate Cancer

Supplementary Table S1. Clinical characteristics for training cohorts.

Variables	Training dataset (n=410)
Age	
<70	373
>=70	37
pT	
<=T2	150
>=T3	255
Unknown	5
pN	
N0	291
N1	67
Unknown	52
GS	
>=8	170
<8	240
BCR	
Yes	48
No	362
Time to BCR	0.90-148.52

Supplementary Table S2. Clinical characteristics for validation cohorts.

Variables	Validation dataset (n=92)
pT	
<=T2	48
>=T3	42
Unknown	2
Surgical margins	
Negative	50
Positive	42
GS	
>=8	15
<8	75
Unknown	2
BCR	
Yes	45
No	47
Time to BCR	0.36-103,43
